# Myocardial infarction with non-obstructive coronary arteries and newly diagnosed cancer: is there a link? Case report

**DOI:** 10.1093/ehjcr/ytag640

**Published:** 2026-08-31

**Authors:** Augustė Ryselytė, Rokas Šerpytis, Sigita Glaveckaitė, Pranas Šerpytis

**Affiliations:** Faculty of Medicine, Vilnius University, M. K. Čiurlionio st. 21, LT - 03101 Vilnius, Lithuania; Vilnius University Hospital Santaros Klinikos, Cardiology and Angiology Center, Santariškių st. 2, LT - 08406 Vilnius, Lithuania; Vilnius University Hospital Santaros Klinikos, Cardiology and Angiology Center, Santariškių st. 2, LT - 08406 Vilnius, Lithuania; Clinic of Cardiovascular Diseases, Institute of Clinical Medicine, Faculty of Medicine, Vilnius University, M. K. Čiurlionio st. 21, LT - 03101 Vilnius, Lithuania; Vilnius University Hospital Santaros Klinikos, Cardiology and Angiology Center, Santariškių st. 2, LT - 08406 Vilnius, Lithuania; Clinic of Cardiovascular Diseases, Institute of Clinical Medicine, Faculty of Medicine, Vilnius University, M. K. Čiurlionio st. 21, LT - 03101 Vilnius, Lithuania; Vilnius University Hospital Santaros Klinikos, Cardiology and Angiology Center, Santariškių st. 2, LT - 08406 Vilnius, Lithuania; Clinic of Cardiovascular Diseases, Institute of Clinical Medicine, Faculty of Medicine, Vilnius University, M. K. Čiurlionio st. 21, LT - 03101 Vilnius, Lithuania

**Keywords:** Myocardial infarction with non-obstructive coronary arteries, Renal cell carcinoma, Cardiovascular magnetic resonance, Computed tomography, Multidisciplinary approach, Case report

## Abstract

**Background:**

Although patients can present with classical myocardial infarction (MI) symptoms requiring urgent evaluation, a subset exhibits no significant obstructive lesions on coronary angiography, revealing a distinct clinical entity known as MI with non-obstructive coronary arteries. This report presents a novel case of a 66-year-old woman with MI with non-obstructive coronary arteries and newly diagnosed renal cell carcinoma. We discuss possible pathophysiological mechanisms that may contribute to the coexistence of these conditions, including systemic inflammation, endothelial dysfunction, oxidative stress, hypercoagulability, and coronary vasospasm.

**Case summary:**

The patient presented with acute chest pain radiating to the left arm, dyspnoea, elevated high-sensitivity troponin I (319–2114 ng/L), and mild ST depressions in V4–V6. Invasive coronary angiography showed no significant changes leading to a diagnosis of MI with non-obstructive coronary arteries. A cardiovascular magnetic resonance was performed and detected local hypokinesis in the inferoseptal and inferior left ventricular walls without a scar. Accidentally, a mass in the left kidney was observed. An abdominal ultrasound and subsequent computed tomography confirmed the diagnosis of renal cell carcinoma in the lower pole of the left kidney without distant metastasis. A multidisciplinary discussion led to a successful laparoscopic nephrectomy and remission.

**Conclusion:**

This clinical case exemplifies the multifaceted nature of clinical presentations and highlights the importance of considering non-cardiac causes in the differential diagnosis of MI with non-obstructive coronary arteries. Shared risk factors, diagnostic complexities, and treatment considerations necessitate a multidisciplinary approach involving cardiology, nephrology, and oncology for optimal patient care.

Learning points

- MINOCA remains a working diagnosis that requires systematic evaluation to identify the underlying mechanism and exclude alternative causes of myocardial injury.
- Occult non-cardiac conditions, including malignancy, should be considered in selected patients with MINOCA when the underlying aetiology remains uncertain, highlighting the importance of multidisciplinary collaboration in complex clinical presentations.

## Primary specialties involved other than cardiology

Oncology, nephrology

## Introduction

Myocardial infarction (MI) with non-obstructive coronary arteries (MINOCA) is a clinical syndrome characterized by evidence of MI without significant coronary artery disease (CAD) on angiography, defined as stenosis severity of ≤50% in major epicardial coronary arteries in the absence of alternative explanations for the clinical presentation of myocardial injury.^1^ Although first described in the early 20th century, MINOCA gained substantial scientific attention only in recent decades.^2^ It is now increasingly recognized, accounting for approximately 5–10% of acute MI cases undergoing coronary angiography.^3^ Despite its growing recognition, MINOCA remains diagnostically and therapeutically challenging due to its heterogeneous aetiology. Clinicians must consider a broad differential diagnosis, including coronary plaque disruption, spontaneous coronary artery dissection, epicardial coronary artery spasm, coronary microvascular dysfunction, Takotsubo cardiomyopathy, myocarditis, coronary thromboembolism, various forms of type 2 MI, and cases of undetermined origin.^4^ Endothelial dysfunction and systemic inflammation are central to the pathophysiology of MINOCA, promoting vascular instability, nitric oxide (NO) depletion, and a prothrombotic milieu. These mechanisms are particularly relevant in systemic inflammatory conditions, such as eosinophilic granulomatosis with polyangiitis, or malignancies like cancer, where persistent inflammation and pro-inflammatory cytokine activity may worsen microvascular dysfunction.^5,6^ This report presents a clinical case of MINOCA in the context of newly diagnosed renal cell carcinoma (RCC), accompanied by a review of the current literature discussing potential mechanisms that may contribute to myocardial injury in patients with malignancy. The case highlights the diagnostic challenges of MINOCA, emphasizes the importance of considering underlying non-cardiac conditions when the aetiology remains uncertain, and illustrates the value of a multidisciplinary management approach involving cardiology, nephrology, and oncology.

## Summary figure

**Figure:**
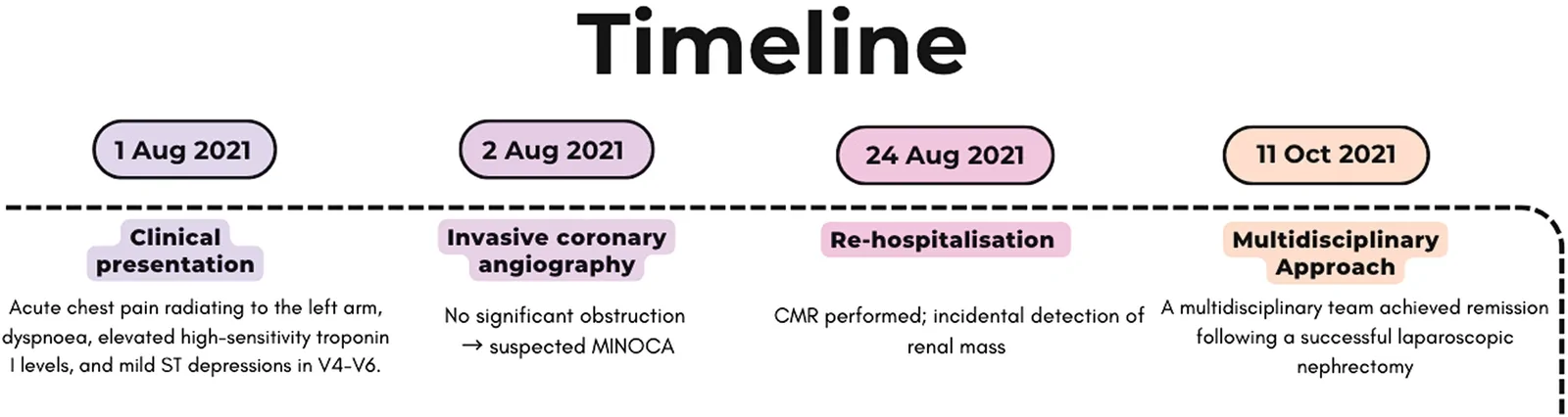


## Case presentation

**Figure 1:**
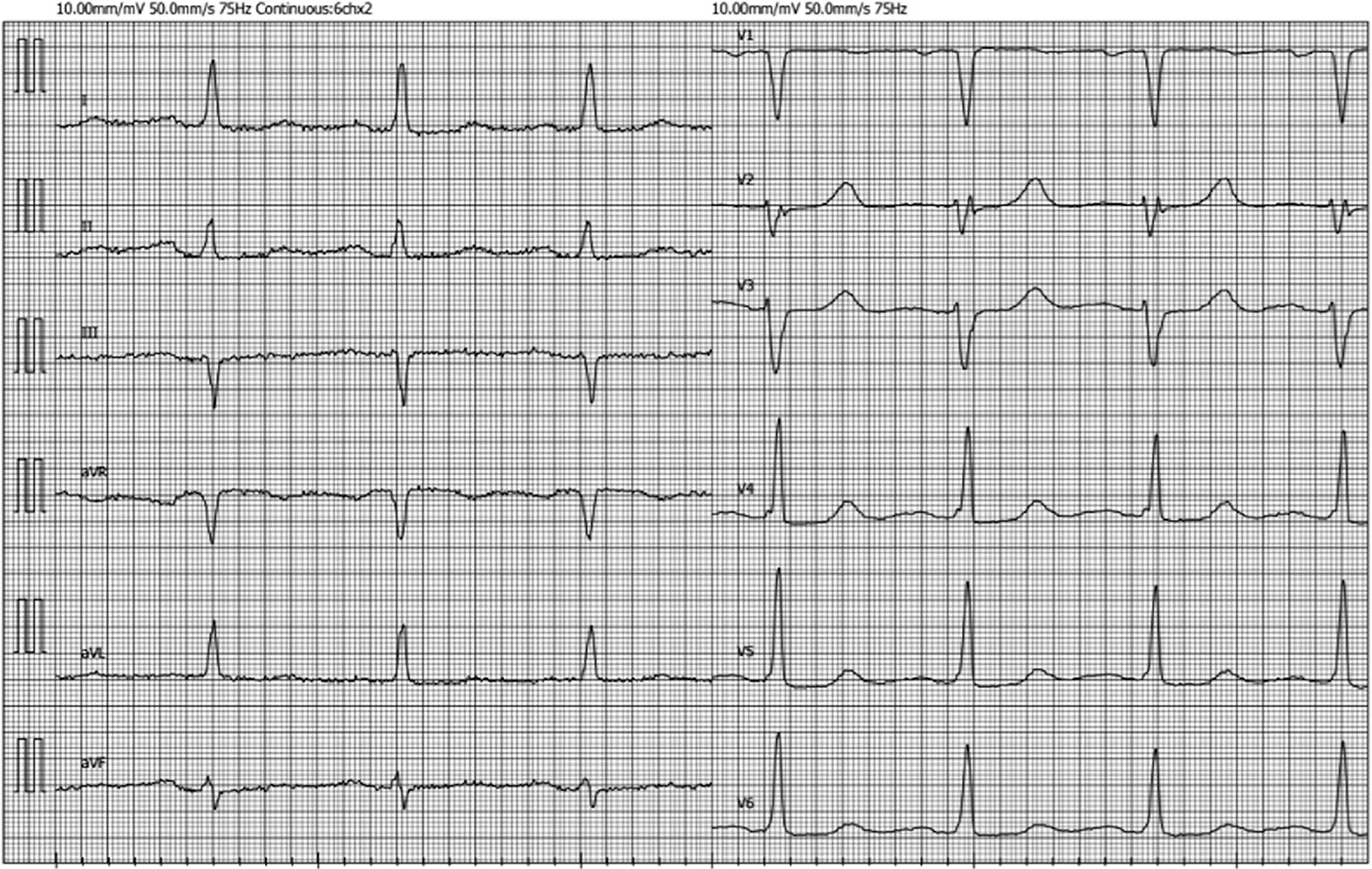
ECG on the admission representing sinus rhythm 72 b.p.m. with mild ST depressions in the precordial leads V4—V6.

**Figure 2:**
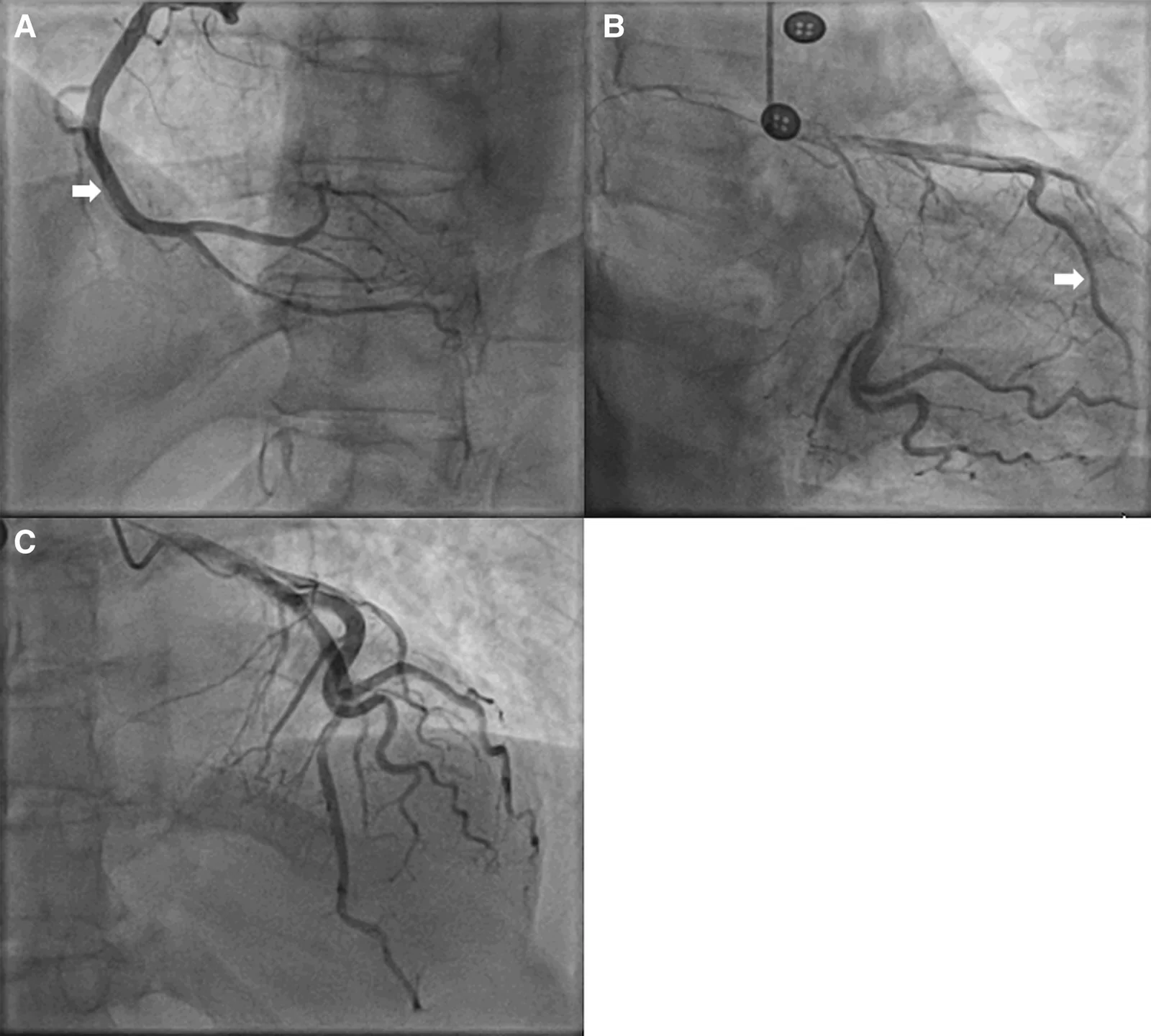
Invasive coronary angiography representing 20% of stenoses in the right coronary artery S2 (*A*, arrow) and left anterior descending artery S7 (*B*, arrow) segments. Left main, including the circumflex artery with no visible significant stenosis (*C*).

A 66-year-old woman with a known history of hypertension, dyslipidemia, and hypothyroidism was admitted to the hospital due to a sudden onset of chest pain while dancing the waltz, which radiated to her left arm and was accompanied by shortness of breath. The pain endured for approximately 30 min. The patient had a history of lumbar bilateral radiculopathy due to lumbosacral intervertebral disc herniations. This condition had resulted in mild paresis of her right foot. The patient was not taking any regular medication due to non-adherence. The initial diagnostic workup included several key investigations. High-sensitivity troponin I levels were elevated, increasing from 319 ng/L at presentation to a peak of 2114 ng/L within a few hours, followed by a decline to 1273 ng/L at discharge, confirming a dynamic rise-and-fall pattern consistent with myocardial injury. The electrocardiogram (ECG) demonstrated normal sinus rhythm at 72 b.p.m., with mild ST-segment depressions observed in the precordial leads V4–V6 (*Figure 1*). Invasive coronary angiography revealed non-obstructive coronary artery disease, with up to 20% stenoses identified in the right coronary artery (segment S2) and the left anterior descending artery (segment S7), consistent with the diagnostic criteria for MINOCA (*Figure 2*). Transthoracic echocardiography showed no significant abnormalities. It should be noted that intracoronary imaging with intravascular ultrasound (IVUS) or optical coherence tomography (OCT), as well as coronary vasospasm provocation testing, were not performed during the index hospitalization. Additional laboratory investigations relevant to the MINOCA evaluation showed normal D-dimer levels (135 μg/L), normal platelet and leukocyte counts, low inflammatory activity (C-reactive protein 2.11 mg/L at presentation, increasing slightly to 3.94 mg/L before discharge), and a normal B-type natriuretic peptide concentration (36.8 ng/L). Erythrocyte sedimentation rate (ESR) was not routinely assessed during the index hospitalization. Medical therapy was initiated, including aspirin as a single antiplatelet agent, a beta-blocker, an angiotensin-converting enzyme (ACE) inhibitor, and a statin, in order to reduce the risk of recurrent cardiovascular events.

**Figure 3:**
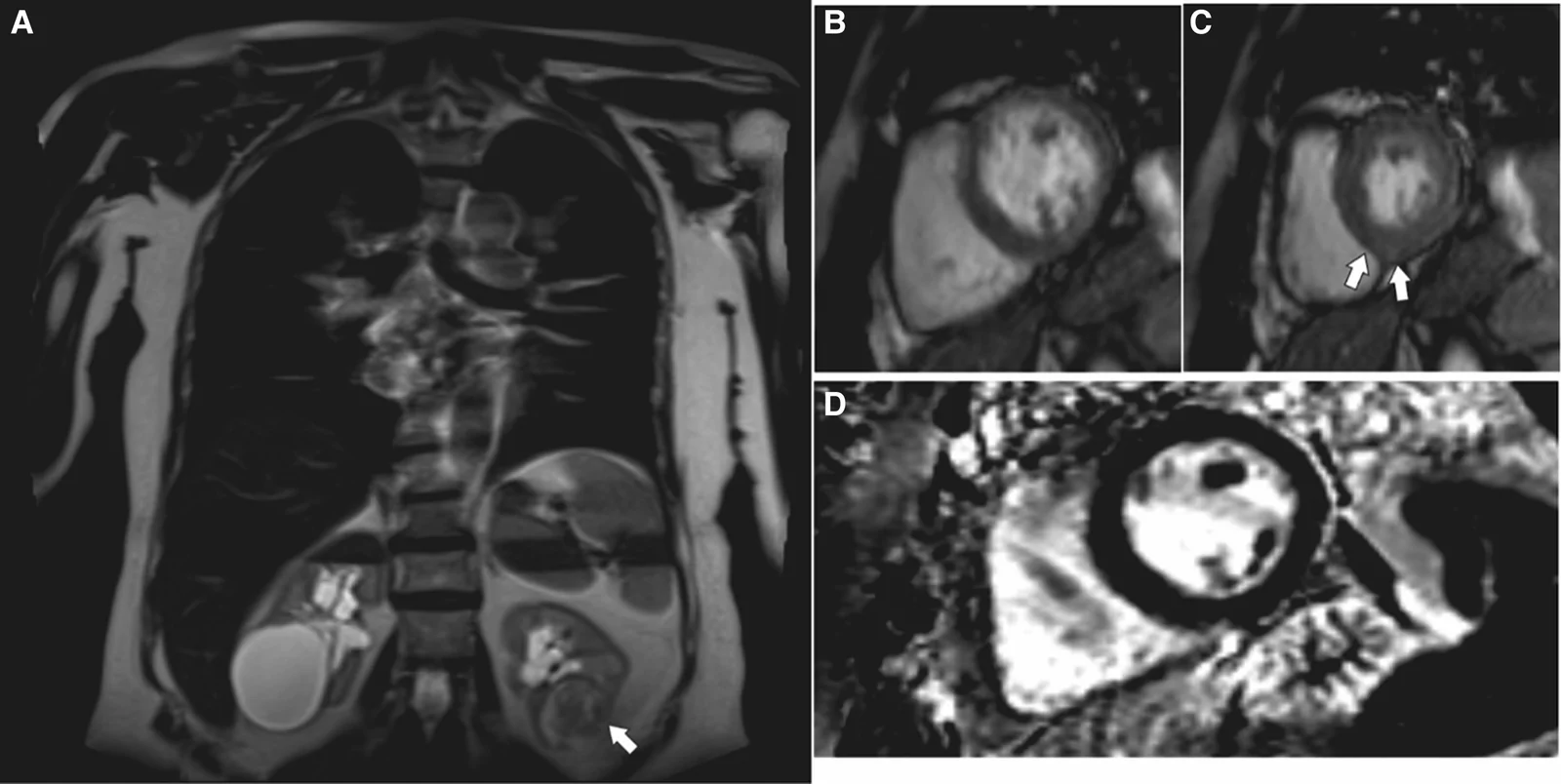
Cardiovascular magnetic resonance scan showing an irregular mass in the lower pole of the left kidney on the frontal localizer view (*A*, arrow), regional hypokinesia of the inferoseptal and partially inferior wall at mid-ventricular level on cine images (*B*—diastole; *C*—systole, arrows), and absence of late gadolinium enhancement in the corresponding short-axis image (*D*).

**Figure 4:**
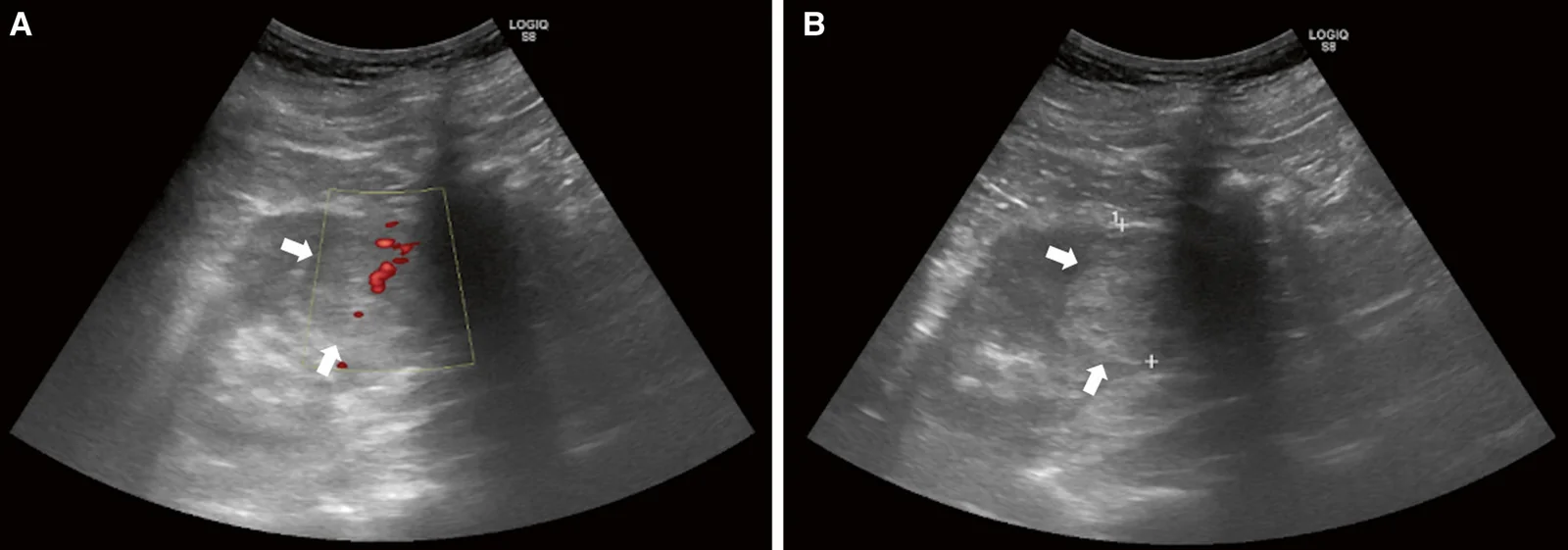
Abdominal ultrasound images showing the presence of vascular flow within an isoechogenic mass at the lower pole of left kidney using colour Doppler (*A*, arrows), and an isoechogenic mass without colour Doppler (*B*, arrows).

**Figure 5:**
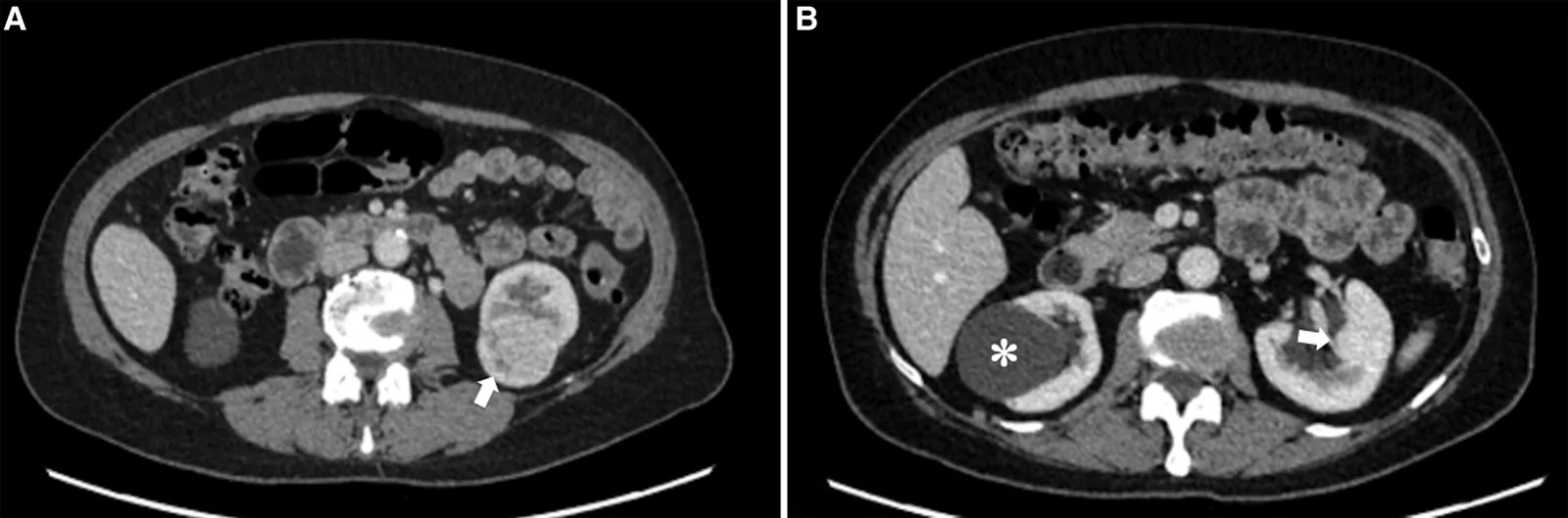
Axial contrast-enhanced CT images demonstrating a dorsally located mass measuring up to 48 mm in the lower pole of the left kidney (*A*, arrow), visualized again in a more cranial slice (*B*, arrow), together with a right renal parenchymal cyst measuring up to 57 mm (*B*, asterisk).

Twenty days after the index event, the patient presented with shortness of breath on moderate-intensity physical activity, weight loss of 2 kg in a month, and night sweats. Troponin I level had normalized to 4 ng/L. The patient underwent cardiac magnetic resonance imaging (CMR), although ideally recommended within 7 days (maximum 14 days) post-event, logistical constraints delayed the imaging. The CMR scan with T2-weighted short tau inversion recovery and T2 mapping did not show signs of myocardial oedema. However, the scan detected local hypokinesis in inferoseptal and inferior walls at midventricular level without a scar, in the absence of late gadolinium enhancement. The adenosine stress perfusion test was negative for ischaemia. Incidentally, CMR also revealed an irregular mass located at the lower pole of the left kidney, measuring up to 44 mm in diameter (*Figure 3*). An abdominal ultrasound, followed by a computed tomography (CT) scan, confirmed the presence of a RCC in the lower pole of the left kidney. The tumour was classified as clear cell type, Grade III according to the ISUP/WHO system, with a pathological stage of pT3a, indicating a 4.7 cm mass extending into the perinephric fat and renal vein branches. Lymphovascular invasion (LVI-3) was also present, but no distant metastases were identified (*Figures 4* and *5*). Simultaneously, the identification of kidney cancer changed the therapeutic approach. The case was comprehensively reviewed by a multidisciplinary team involving cardiologists, nephrologists, and oncologists, ensuring a cohesive, patient-focused treatment strategy. Alongside ongoing cardiac management, the patient underwent a successful laparoscopic nephrectomy for removal of the kidney tumour. Perioperative antithrombotic management was individualized according to surgical recommendations. Aspirin was discontinued 5 days before nephrectomy, prophylactic low-molecular-weight heparin was administered during the perioperative period, and the patient subsequently continued aspirin 100 mg once daily as long-term secondary cardiovascular prevention. The perioperative course was uneventful, with stable hemodynamics and no cardiovascular complications observed during or after the nephrectomy. Due to the localized nature and complete surgical resection of the tumour without distant metastasis, the oncology team opted for active surveillance, postponing systemic anticancer therapies such as tyrosine kinase inhibitors or immune checkpoint inhibitors to avoid potential cardiovascular complications. Comprehensive follow-up, including imaging and laboratory assessments at scheduled intervals, was implemented to detect potential recurrence or metastasis. The ECGs remained stable, showing normal sinus rhythm without ischaemic changes. Follow-up transthoracic echocardiography performed approximately 10 months after nephrectomy demonstrated a preserved left ventricular ejection fraction (>55%) without reported regional wall motion abnormalities. No follow-up CMR examination or global longitudinal strain (GLS) assessment was performed. Long-term oncological follow-up included serial thoracoabdominal CT examinations, which demonstrated no evidence of local recurrence or distant metastatic disease during the available follow-up period. During follow-up, the patient remained asymptomatic from a cardiovascular perspective, haemodynamically stable, and in oncological remission.

## Discussion

In 2017, the European Society of Cardiology (ESC) first established diagnostic criteria for MINOCA, based on the third Universal Definition of Myocardial Infarction.^7^ The third Universal Definition was updated to the fourth in 2018, refining MINOCA criteria to include only ischaemic mechanisms and excluding non-ischaemic causes such as Takotsubo cardiomyopathy, myocarditis, and non-ischaemic cardiomyopathy.^8^ Research indicates that MINOCA is more prevalent in women and younger individuals compared to acute MI with obstructive CAD, emphasizing the importance of considering sex-specific and age-specific factors in the assessment and treatment of MINOCA patients. Recent data further highlight significant sex-related differences. Women aged ≤70 years with MINOCA exhibit a higher incidence of major adverse events compared to men, likely attributable to distinct pathophysiological mechanisms driving ischaemic events in women.^9^ Studies indicate that MINOCA patients generally have a more favourable prognosis compared to those with obstructive CAD, with lower mortality rates and lower major adverse cardiac events.^10^ In the present case, the diagnosis of MINOCA was established based on a clinical presentation consistent with acute coronary syndrome, a dynamic rise and fall in cardiac troponin levels, non-obstructive coronary arteries on angiography, and no overt alternative explanation at the time of initial evaluation, in line with contemporary ESC recommendations.^4^ However, as emphasized in current guidelines, MINOCA represents a working diagnosis that requires further aetiological clarification.^4^ Beyond coronary angiography and CMR imaging, intravascular imaging techniques such as IVUS and OCT may provide important additional diagnostic information in the evaluation of MINOCA cases.^11^ Importantly, in the present case, CMR was performed beyond the 7–14-day window recommended by current guidelines, which may have reduced the sensitivity for detecting late gadolinium enhancement. In addition, intracoronary imaging (IVUS/OCT) was not performed, representing a further limitation.

Renal cell carcinoma, the most common malignant kidney tumour, originates in the renal tubular epithelium and constitutes over 90% of adult renal neoplasms. Men are 1.5 times more likely than women to develop RCC, with the highest incidence occurring between the ages of 60 and 70.^12^ Renal cell carcinoma is often diagnosed incidentally in an advanced stage, emphasizing the importance of early detection and screening programmes to improve patient outcomes.^12^ Understanding the epidemiology and risk factors associated with RCC is crucial for effective management and treatment strategies.

The coexistence of MINOCA and RCC remains poorly understood and may be related to shared pathophysiological mechanisms and overlapping cardiovascular risk factors. Emerging evidence suggests that malignancy-associated systemic inflammation may impair coronary microvascular function and promote vasospasm, providing a potential mechanistic link between RCC and MINOCA. Both conditions involve systemic inflammation, endothelial dysfunction, oxidative stress, and a hypercoagulable state.^5^ Cardiovascular disease and RCC also share several overlapping risk factors, including genetic predisposition, polycythaemia, hypertension, obesity, smoking, and metabolic disorders such as hyperglycaemia which may partially explain their coexistence in the present case.^5^

The pro-inflammatory state associated with RCC may impair coronary microvascular endothelial function and has been proposed as a potential contributor to mechanisms implicated in MINOCA.^5^ Renal cell carcinoma activates inflammatory signalling pathways, including c-Jun N-terminal kinase and p38 mitogen-activated protein kinase, resulting in elevated production of pro-inflammatory cytokines and chemokines.^13^ A key inflammatory marker, interleukin-6 (IL-6), is pivotal in endothelial dysfunction. Elevated IL-6 levels are associated with increased vascular permeability and atherosclerosis.^14^

Renal cell carcinoma has been associated with a prothrombotic state that may contribute to mechanisms implicated in MINOCA; however, such a mechanism could not be demonstrated in the present case.^5^ In a large cohort study of RCC patients, cardiovascular disease accounted for over 28% of all deaths, with heart disease emerging as the leading cause of mortality beyond 5 years after diagnosis, underscoring the long-term cardiovascular risks associated with RCC.^15^

Tumour-derived procoagulants, such as microparticles and cancer procoagulants, activate coagulation cascades, increasing thrombin and fibrin formation.^5^ This mechanism is particularly relevant in RCC, as the tumour microenvironment enhances the release of microparticles shed by tumour cells, which carry inflammatory mediators, amplifying the coagulation response and worsening endothelial dysfunction. In the current report, standard laboratory markers of hypercoagulability (including D-dimer and coagulation parameters) were assessed and found to be within normal limits. This further supports the absence of a clearly demonstrable systemic prothrombotic state in this patient; however, an extended thrombophilia work-up was deferred following the prompt identification of the underlying malignancy.

Additionally, inflammatory mediators including tumour necrosis factor-alpha (TNF-α) upregulate tissue factor and plasminogen activator inhibitor-1, further amplifying coronary thrombosis and embolism risks.^14^ The interaction between IL-6 and TNF-α plays a crucial role in the pathophysiology of RCC-associated cardiovascular complications, as both cytokines contribute to oxidative stress, enhance endothelial dysfunction, and increase the risk of vascular and thrombotic events.^5,14^

In addition to its systemic effects, RCC has been hypothesized to promote mechanisms that may favour coronary vasospasm and increased myocardial oxygen demand, although these mechanisms were not specifically evaluated in the present case. Cancer-induced systemic inflammation and oxidative stress stimulate the production of potent vasoconstrictors, such as endothelin-1 (ET-1) and catecholamines, which sensitize coronary vascular smooth muscle to constriction. Elevated ET-1 levels enhance vascular contractility, while catecholamines activate α-adrenergic receptors, further promoting vasospasm.^16^

Renal cell carcinoma therapies, mainly targeted therapies and immunotherapies, have revolutionized the management of advanced disease but may increase the risk of cardiovascular complications, including MINOCA, by exacerbating inflammatory pathways. Targeted therapies, such as multikinase inhibitors (MKIs; e.g. sorafenib, sunitinib, and axitinib) and mammalian target of rapamycin inhibitors, disrupt tumour angiogenesis and growth pathways. However, these agents can elevate inflammatory cytokines, impair endothelial function, reduce NO bioavailability, and promote vasoconstriction, potentially increasing blood pressure and cardiovascular risk.^17^ Additionally, combining MKIs with immune checkpoint inhibitors (e.g. nivolumab and ipilimumab) can further heighten inflammatory burden, contributing to immune-related adverse cardiac events such as myocarditis, endothelial dysfunction, and thrombosis.^18^ Anaemia, prevalent in RCC patients, can indirectly elevate myocardial oxygen demand, further contributing to ischaemic risks through mechanisms involving chronic inflammation and iron deficiency.^19^ Furthermore, drug–drug interactions may impact cardiovascular management. Antihypertensive medications often required in RCC patients receiving MKIs should be carefully selected, especially considering potential interactions with CYP3A4 inhibitors like diltiazem or verapamil, which may affect MKI metabolism.^17,20^ Careful cardiovascular management in RCC patients must also consider potential interactions between cardiovascular medications and cancer treatments, as well as renal impairment and associated comorbidities.

A multidisciplinary cardio-oncology approach is crucial to effectively integrate cancer treatment with cardiovascular care, ensuring comprehensive management tailored to the needs of patients diagnosed with MINOCA and RCC. These patients often present with overlapping clinical manifestations, including chest pain, fatigue, and dyspnoea, complicating timely diagnosis and potentially delaying optimal treatment strategies.^5^ A dedicated cardio-oncology team—typically comprising cardiologists, oncologists, nephrologists, specialized nurses, and other healthcare professionals—collaborates closely to navigate these complexities, particularly when managing RCC patients undergoing treatments with substantial cardiovascular implications.

A significant challenge faced by the cardio-oncology team involves the management of anticoagulation in RCC patients at elevated bleeding risk. This requires careful balancing of thrombotic vs. haemorrhagic risks, especially in patients treated with agents such as MKIs, which can exacerbate coagulopathy. To mitigate treatment-associated cardiovascular complications, structured cardiovascular monitoring—including regular echocardiography, electrocardiography, and cardiac biomarker assessments—should be conducted before, during, and after oncological therapies. Additionally, targeted lifestyle interventions, encompassing dietary adjustments and structured exercise programmes, further contribute to reducing cardiovascular risk. Ultimately, by thoroughly understanding the intricate interactions between oncological treatments and cardiovascular health, the cardio-oncology team can optimize both cardiovascular and cancer outcomes while ensuring patient safety and enhancing quality of life.

## Limitations

Several limitations should be acknowledged. First, intracoronary imaging with IVUS or OCT was not performed, and therefore plaque disruption could not be definitively excluded. Second, coronary vasospasm provocation testing was not undertaken. Third, CMR was performed beyond the time window recommended by current guidelines, which may have reduced its diagnostic sensitivity. In addition, no follow-up CMR or GLS assessment was available to further characterize the evolution of myocardial abnormalities. Consequently, the precise mechanism underlying MINOCA in this patient remains uncertain. Although RCC may represent a potential contributing factor, the present case does not establish a causal relationship and should be regarded as a hypothesis-generating observation.

## Conclusion

This case highlights the importance of considering non-cardiac causes, including malignancy, in patients presenting with MINOCA, particularly when the underlying aetiology remains unexplained. While shared pathophysiological mechanisms—including systemic inflammation, oxidative stress, endothelial dysfunction, hypercoagulability, and coronary vasospasm—have been proposed as potential contributors to myocardial injury in patients with malignancy, these mechanisms were not directly demonstrated in the present case.

Accordingly, establishing causality in a single case report remains inherently limited. Therefore, this case should be interpreted as hypothesis generating, suggesting a possible association between RCC and MINOCA while underscoring the need for careful evaluation of underlying, potentially occult conditions in such clinical scenarios.

A multidisciplinary cardio-oncology approach—integrating cardiologists, oncologists, nephrologists, and specialized nurses—is essential for optimal patient management, enabling a balanced strategy between oncological treatment, cardiovascular monitoring, and individualized risk reduction. Increased awareness of these complex interactions and close interdisciplinary collaboration may contribute to improved diagnostic accuracy, patient outcomes, and overall quality of care in patients presenting with MINOCA and concomitant malignancy.

## Lead author biography


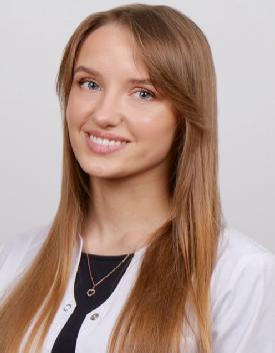


I am Augustė Ryselytė, medical doctor, a resident doctor in cardiology. During my medical studies I developed a strong academic and clinical interest in cardiology and actively pursued opportunities to deepen my knowledge through research and clinical training. I am particularly drawn to interdisciplinary collaboration and case-based learning, which I believe are essential for improving patient care. As a cardiology resident, I remain highly motivated to further expand my academic expertise and contribute to the advancement of the field.

## Data Availability

The data presented in this study are available on request from the corresponding author.
